# Fungal Pathogenesis-Related Cell Wall Biogenesis, with Emphasis on the Maize Anthracnose Fungus *Colletotrichum graminicola*

**DOI:** 10.3390/plants11070849

**Published:** 2022-03-23

**Authors:** Alan de Oliveira Silva, Lala Aliyeva-Schnorr, Stefan G. R. Wirsel, Holger B. Deising

**Affiliations:** Chair for Phytopathology and Plant Protection, Institute for Agricultural and Nutritional Sciences, Faculty of Natural Sciences III, Martin-Luther-University Halle-Wittenberg, Betty-Heimann-Str. 3, D-06120 Halle (Saale), Germany; alan.de-oliveira-silva@landw.uni-halle.de (A.d.O.S.); lala.aliyeva-schnorr@landw.uni-halle.de (L.A.-S.); stefan.wirsel@landw.uni-halle.de (S.G.R.W.)

**Keywords:** appressorium, β-glucan, cell wall biogenesis, chitin, chitosan, evasion of PAMP-triggered immunity, hemibiotrophy, melanin, pathogen-associated molecular patterns

## Abstract

The genus *Colletotrichum* harbors many plant pathogenic species, several of which cause significant yield losses in the field and post harvest. Typically, in order to infect their host plants, spores germinate, differentiate a pressurized infection cell, and display a hemibiotrophic lifestyle after plant invasion. Several factors required for virulence or pathogenicity have been identified in different *Colletotrichum* species, and adaptation of cell wall biogenesis to distinct stages of pathogenesis has been identified as a major pre-requisite for the establishment of a compatible parasitic fungus–plant interaction. Here, we highlight aspects of fungal cell wall biogenesis during plant infection, with emphasis on the maize leaf anthracnose and stalk rot fungus, *Colletotrichum graminicola*.

## 1. Differentiation of Fungal Infection Structures and Expression of a Hemibiotrophic Lifestyle

Over 600 species belonging to the genus *Colletotrichum* (Sordariomycetes, Ascomycota) cause disease in more than 3200 monocot and dicot plants species, and cause dramatic yield losses in several crops [1]. Estimates indicate that one of the best studied pathogens of this genus, i.e., the causal agent of leaf anthracnose and stalk rot of maize, *Colletotrichum graminicola*, causes annual losses of approximately one billion dollars in the United States alone [2]. In order to gain entry into their hosts, the vast majority of *Colletotrichum* species differentiates melanized invasive cells called appressoria or hyphopodia on the plant surface, which generate high turgor pressure to breach cuticle and epidermal plant cell wall. After invasion, infection structures that are successively differentiated show biotrophic and necrotrophic lifestyles, and this hemibiotrophic lifestyle is characteristic of most *Colletotrichum* species [1,3,4]. However, the morphology of infection structures and the period of biotrophy vary considerably across species or species complexes. For example, several species belonging to the *Colletotrichum destructivum* complex, including *C. destructivum*, *C. tabacum*, and *C. higginsianum*, form a biotrophic lobed infection vesicle and primary hyphae that are strictly confined to the first infected cell, and hence the term localized hemibiotrophy is used to describe this type of hemibiotrophy. Subsequently, primary hyphae give rise to narrow and destructive secondary hyphae that mark the onset of the necrotrophic phase of infection, and initiate colonization of the host tissue. By contrast, species belonging to the *Colletotrichum graminicola* complex such as *C. graminicola* and *C. sublineolum*, form a globular biotrophic infection vesicle immediately after invading the epidermal host cell, from which voluminous filamentous primary hyphae develop. In species of the graminicola complex, biotrophic hyphae are not confined to the first infected epidermal cells, but invade neighboring epidermal or mesophyll cells primarily through plasmodesmata, and a so-called extended hemibiotrophic lifestyle is established [3,4,5]. Necrotrophic hyphae emerge from several primary hyphae, colonize the plant cells, and cause massive tissue destruction [6,7]. Importantly, in most species of the graminicola complex, formation of biotrophic and necrotrophic hyphae is poorly synchronized, so that hyphae differing in function and morphology occur side by side in tissue samples, severely hampering the association of biochemical and molecular alterations with a defined pathogenic or nutritional lifestyle.

An aspect of critical importance in the infection biology of *Colletotrichum* species relates to how defense responses are circumvented at the moment of host invasion. Regardless of the duration of biotrophy and whether biotrophic hyphae occur only in the first host cell or spread to neighboring cells, infection vesicles and primary hyphae are tightly surrounded by the plasma membrane of their living host cell(s). The close physical contact of the fungal cell wall and the plant plasma membrane, which had been invaginated by the pathogen, is comparable to that established by haustoria differentiated by obligate biotrophs such as rust fungi [8,9,10]. Plants constitutively secrete low levels of enzymes cleaving fungal cell wall polymers, and mobile pathogen-associated molecular patterns (PAMPs) are thus generated in close proximity to the plant plasma membrane, which is spiked with PAMP receptors. A key question of understanding the establishment of a compatible pathogenic interaction is therefore how the pathogen is able to circumvent PAMP perception and suppress plant defense response initiation. Conceivably, infection stage-specific adaptation of the surface composition of fungal infection structures, including secretion of apoplastic effector proteins that bind to and mask specific surface carbohydrate polymers, play prime roles in the *Colletotrichum* infection process. As the maize pathogen *C. graminicola* has been established as a model pathogen during recent years, this review will focus on this fungus, with side-views on other *Colletotrichum* and non-*Colletotrichum* pathogens to highlight the variability of infection strategies.

## 2. Fungal Structural Cell Wall Polymers Are a Rich Source of PAMPs

Comparison of the composition of cell walls of yeasts, dimorphic and filamentous fungi such as the ascomycetes *Saccharomyces cerevisiae*, *Schizosaccharomyces pombe*, *Candida albicans*, *Aspergillus fumigatus*, *Neurospora crassa*, and of the dimorphic basidiomycete fungus *Cryptococcus neoformans* reveals that polymeric chitin, chitosan, β-1,3-glucan, β-1,6-glucan, mixed β-1,3-/β-1,4-glucan, α-1,3-glucan, galactomannan, melanin, and glycoproteins represent major constituents [11,12,13,14,15,16,17]. Detailed chemical analyses of cell walls of the opportunistic mammalian pathogen, *A. fumigatus*, which is the best studied filamentous fungus with respect to cell wall chemistry and architecture, revealed that 40% of the dry cell wall material is alkali-insoluble and composed of polysaccharides (>90%), with 20–35% linear β-1,3-glucan carrying 4% β-1,6-links. In this fungus, β-1,6-glucosidic bonds only occur as branch points in β-1,3-glucan chains, leading to a three-dimensional network with non-reducing ends, to which chitin, galactomannans, and β-1,3/1,4-glucans are covalently anchored. The alkali-insoluble cell wall matrix, thus represents a large heteropolymeric cell wall carbohydrate complex [11,13,16] and determines wall structure and shape of the cell. In contrast to *A. fumigatus*, β-1,6-glucan represents a major cell wall polymer in *S. cerevisiae* and in the dimorphic mammalian pathogens *C. albicans* and *C. neoformans* [18,19].

Pioneering work deciphered cell wall biosynthesis in *Schizophyllum commune*. Autoradiography showed synthesis of alkali-insoluble chitin and glucan polymers at the hyphal apex, and consecutive introduction of covalent links between cell wall polymers at the sub-apex [20,21]. In agreement with these early discoveries, Latgé and co-workers suggested that, in *A. fumigatus*, cell wall biogenesis begins with the formation of individual polysaccharides such as β-1,3-glucan, β-1,3/1-4 mixed linked glucan, chitin, and galactomannan, which is followed by branching of β-1,3-glucan, increasing the number of acceptor sites, and finally ends with covalently connecting chitin, galactomannan, β-1,3-glucan, and β-1,3/1,4-glucan with the acceptor sites at glucan branches [13]. In addition to carbohydrates, cell wall-bound proteins may account for up to 10% of the fungal cell wall, and more than half of the major cell wall proteins are post-translationally modified by the addition of a glycosylphosphatidylinositol (GPI) anchor [14]. In yeast, cell wall-bound glycoproteins were found linked to β-1,6-glucan. These proteins are originally anchored to the membrane via their GPI-residue, but subsequently cleaved and transferred onto β-1,6-glucan, using the sugar moiety of GPI as a bridge [13,22,23,24]. Due to their use in covalently linking the structural cell wall polymers, GPI-anchored proteins can be regarded as structure-determining molecules of the fungal cell wall.

The alkali-soluble cell wall polymer fraction may play only indirect roles in cell wall structure, as these compounds may act as mucosal determinants of cell wall pore sizes and mobility of cell wall-remodeling enzymes. Moreover, such compounds may be secreted onto the cell surface to cover those cell wall polymers that may act as pathogen-associated molecular patterns (PAMPs). Secretion of mucosal polymers could thus contribute to masking of plant-invading hyphae [25,26] (see below).

A comparison of cell walls of fungi differing in taxonomy, morphology, and lifestyle highlights an enormous variability in fungal cell wall composition and organization [14]. Distinct cell wall polymers potentially acting as PAMPs have been identified and, increasing the complexity of cell wall structure, their synthesis and/or surface exposure may differ in a cell type-specific manner. For example, as plants constitutively synthesize chitinases or β-glucanases, chitin and β-glucan fragments may be liberated from fungal cell walls and, in turn, interact with pattern recognition receptors (PRRs) in the plant plasma membrane, activate defense responses, and lead to PAMP-triggered immunity (PTI). Presumably, cell wall polymer synthesis and surface exposure may differ across infection hyphae, depending on their lifestyle. Alternatively, some of the fungal secreted proteins called apoplastic effectors may exhibit specific carbohydrate-binding properties, mask the hyphal surface, and interfere with PTI. As a third option of circumventing plant defense responses, a PAMP-releasing polymer or the PAMP itself may be enzymatically modified to evade cleavage by hydrolytic plant enzymes or detection by receptors of the host. This brief outline underlines that the composition of the fungal cell wall represents a key factor in pathogenesis [27].

In this article, we will provide examples for each of the above-mentioned mechanisms of cell wall synthesis and/or re-modeling that promote the compatibility between pathogen and host.

## 3. Cell Wall Polymers Are Major Determinants in the *C. graminicola* Infection Process

Interestingly, fungi belonging to different taxa have developed highly specialized infection structures, called appressoria, in order to breach the outer cell wall of the host plant and to gain access to nutrients in the plants’ cells [8]. Already in the first issue of the journal ‘Berichte der Deutschen Botanischen Gesellschaft’, as published in 1883, Frank reported on a new disease of garden beans caused by a fungus called *Gloeosporium lindemuthianum* [28], and even today this fungus, re-named as *Colletotrichum lindemuthianum*, is an economically relevant pathogen. Frank’s observation of infection-related morphogenesis on the plant cuticle, and discovery of an adhesion organ what he called ‘appressorium’ was not surprising due to strong appressorial melanization occurring in basically all *Colletotrichum* species. Frank noticed that appressoria formed on the surface of bean fruits, but not on glass, and recognized the penetration pore at the appressorial base as the point of fungal invasion into the host tissue. He concluded that appressoria are specific ‘organs’ required for plant invasion. Hence, almost 140 years ago the function of fungal appressoria had been proposed and, in addition, infection-related cell wall modification in form of melanization had been reported.

### 3.1. In Colletotrichum Species, Melanin Is Required for Appressorium Function and Plant Invasion

Fungal cell wall-associated melanin is a polymer of 1,8-dihydroxy naphthalene (DHN). Its synthesis is initiated by a polyketide synthase (PKS), forming 1,3,6,8-tetrahydroxynaphthalene (1,3,6,8-THN), which is converted to 1,8-DHN through successive rounds of reduction and dehydration, and DHN is oxidatively cross-linked in a radical reaction initiated by phenoloxydases such as laccases [29]. Incorporation of melanin into hyphal cell walls has been reported for several fungal species, including pathogens of plants and mammals [29,30,31]. In several fungi, transmission electron microscopy indicated that melanin occurs as an electron-dense outer layer of the cell wall or that it is associated with a matrix outside of the cell wall [29,32]. Thus, as an outer cell wall layer, melanin may be associated with resistance to unfavorable environmental conditions such as UV irradiation, exposure to reactive oxygen species (ROS), desiccation, and hydrolytic enzymes, the latter playing a role in plant infection [29]. Interestingly, conidia of an *A. fumigatus* melanin mutant deficient in the synthesis-initiating PKS, but not conidia of downstream mutants of the DHN biosynthetic pathway, were significantly more susceptible to ROS than wildtype conidia, suggesting that polymerization products of melanin precursors may function as ROS scavengers contributing to virulence in a similar manner as DHN melanin [31,33].

Appressorial melanization, as occurring in *Colletotrichum*, *Magnaporthe*, *Polystigma*, and in *Phyllosticta* species, differs clearly from hyphal cell wall melanization with respect to cell specificity of polymer formation and localization of the product [34,35]. In appressoria, melanin is localized in the cell wall in direct proximity of the appressorial plasma membrane, and not at the outer cell wall layer, arguing against a role as a ROS scavenger. Instead, incrustation of the appressorial carbohydrate polymer network with DHN melanin results in cell wall pore reduction to less than 1 nm and likely changes in mechanical cell wall properties [36,37]. Howard and Ferrari [37] suggested that melanin is required for blocking loss of glycerol, which accumulates in appressoria of *M. oryzae* up to concentrations of 3.2 M, and thus serves as a major osmolyte for generating turgor pressure [38]. Therefore, melanin is considered a vital mechanical component in the penetration process [37].

Employing a method called incipient-cytorrhysis allowed Howard and co-workers to measure the turgor pressure in melanized appressoria of the rice blast fungus, *Magnaporthe oryzae*, and to demonstrate that individual infection cells are able to generate pressure in excess of 8.0 MPa, corresponding to more than 80 bar. Such an enormous turgor pressure is thought to be fully sufficient to breach plant cell walls without major contribution of cell wall-degrading enzymes [39]. The hypothesis that fungal ingress is primarily force-driven was further supported by optical waveguide experiments showing that the turgor of melanized appressoria of *C. graminicola* was translated into force corresponding to some 17 µN, which is exerted at the appressorial base [40,41]. Impressively, if a force of 17 mN μm^−2^ were exerted over the palm of one hand, a human could lift an 8000-kg school bus [42]. This calculation highlights the magnitude of turgor pressure within and force exerted by melanized fungal appressoria, which requires especially fortified cell walls.

**Figure 1:**
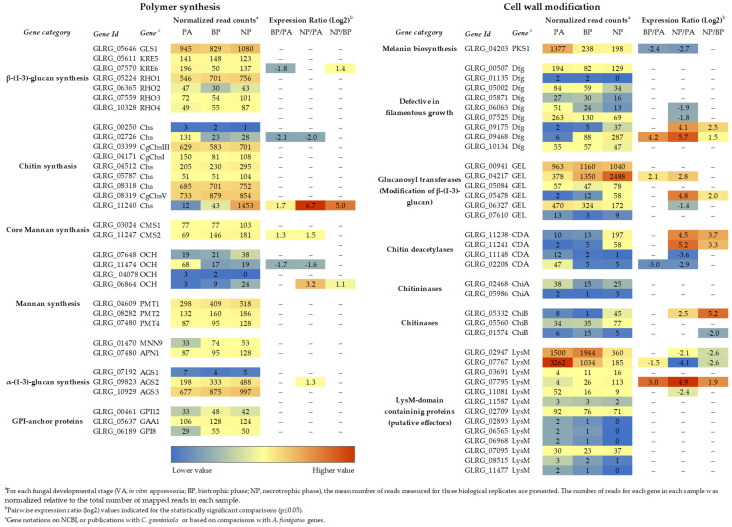
Transcript abundance of cell wall biogenesis genes of *C. graminicola* at different stages of plant infection. ^a^ For each fungal developmental stage (VA, in vitro appressoria; BP, biotrophic phase; NP, necrotrophic phase), the mean number of reads measured for three biological replicates are presented. The number of reads for each gene in each sample was normalized, relative to the total number of mapped reads in each sample. ^b^ Pairwise expression ratio (log2) values indicated for the statistically significant comparisons (*p* ≤ 0.05). ^c^ Gene annotations of NCBI, publications with *C. graminicola*, or based on comparisons with *A. fumigatus* genes. RNA-Seq data are from [3].

The initial enzyme of melanin biosynthesis in *C. graminicola*, Pks1, is required for appressorium melanization, and, plausibly, the corresponding gene *PKS1* is activated in these infection cells (Figure 1) [43]. Like *M. oryzae* mutants lacking melanin, *C. graminicola* Δ*pks1* mutants were unable to invade maize leaves with an intact surface, but were fully virulent on wounded leaves [43]. In contrast to *M. oryzae* appressoria, however, Δ*pks1* mutants of *C. graminicola* showed lateral appressorial germination, occasionally leading to formation of appressorial chains [43], suggesting that melanin serves distinct as well as similar functions in the rice blast and in the maize leaf anthracnose pathogen. Based on the observation that appressoria of melanin-deficient mutants of the cucumber anthracnose pathogen *Colletotrichum lagenarium* (syn. *Colletotrichum orbiculare*) and the bean pathogen *Colletotrichum lindemuthianum* germinated laterally [44,45,46], Kubo and co-workers suggested that melanin is required to determine the direction of penetration peg emergence [47]. Further arguments against the primary role of melanin in osmolyte retainment and turgor generation [37] were provided by a thorough study of appressoria of the Asian soybean rust fungus *Phykopsora pachyrhizi*, which do not melanize. Appressoria of this fungus contain significant concentrations of osmolytes such as the C6-polyol mannitol, C5-polyols, glucose, glycerol, and myo-inositol generated turgor pressure of more than 5 MPa, as shown by incipient cytorrhysis, and penetrated non-biodegradable polytetrafluoroethylene (PTFE) membranes more efficiently than melanized appressoria of *C. graminicola* or *M. oryzae* [48].

Indeed, one may hypothesize that the role of melanin is not to restrict the loss of appressorial osmolytes, but, rather, that this polymer interferes with secretion of cell wall-degrading enzymes such as chitinases or β-1,3-glucanases into the cell wall.

Steady state growth of hyphal filaments is sustained by a continuous flow of secretory vesicles as coordinated by the microtubule and actin cytoskeletons as well as their corresponding motor proteins [49]. Vesicles containing cell wall-synthesizing enzymes accumulate at an apical structure called the Spitzenkörper, before they are directed further to and fuse with the membrane at the hyphal apex. The site of vesicle fusion with the apical plasma membrane and the direction of hyphal growth are determined by the position of the Spitzenkörper [50]. Positioning of the Spitzenkörper, together with transcriptional reprogramming, may thus provide a basis for hyphal morphogenesis, including infection structure differentiation [50]. Appressorium differentiation starts with a loss of hyphal polarity, as indicated by swelling of the germ tube, and the release of secretory vesicles from a Spitzenkörper, which is no longer associated with the hyphal tip, would lead to formation of a dome-shaped or lobed appressorium [8,50,51]. Indeed, staining of germ tube apices of the two rust fungi *Uromyces vignae* and *Puccinia graminis* f. sp. *tritici* with the fluorescence membrane dye FM4-64 membrane showed asymmetric positioning of the Spitzenkörper close to the cell-substratum interface. In agreement with the above hypothesis, the Spitzenkörper, which was clearly visible in germ tubes, disappeared at the onset of appressorium differentiation, and was again observed when the highly polarized penetration peg developed [52]. Because re-initiation of a filamentous penetration hypha at the basis of the appressorium likely requires secretion of cell wall softening hydrolases such as chitinases or β-1,3-glucanases, comparable to initiation of hyphal branches [53], the re-initiation of a Spitzenkörper, as observed in the above-mentioned rust fungi, would be required [52]. One may argue that during appressorium formation, secretory vesicles containing hydrolases, would migrate in a non-directed way to the appressorial plasma membrane and would be secreted into the cell wall. In this scenario, cleavage of cell wall polymers required for controlling the appressorial turgor would be prevented by a melanin layer in the utmost vicinity to the appressorial plasma membrane. Pore size reduction by cell wall melanization to less than 1 nm [36] would restrict the access of chitinases and β-1,3-glucanases to their cell wall substrates and would therefore be indispensable for appressorium function. Indeed, appressoria of Δ*pks1* and Δ*ppt1* mutants of *C. graminicola*, the latter lacking the enzyme 4′-phosphopanthetenyl transferase required for post-translational activation of Pks1, form non-melanized appressoria. These appressoria generate turgor pressure comparable to the WT strain, but disrupt due to cell wall weakening [43,54].

An important question regards the mechanism by which the appressorial basis is kept free of melanin. Interestingly, recent work has shown that at the appressorial base of the rice blast fungus, a septin layer is initially formed and subsequently contracts, yielding a septin ring, which defines the melanin-free penetration pore [55,56]. Thus, in agreement with the hypothesis of Kubo et al. [47], the melanin-deficient penetration pore would allow to re-establish an apical pole, to direct the penetration peg towards the host surface, and to invade the host epidermal cell.

**Figure 2:**
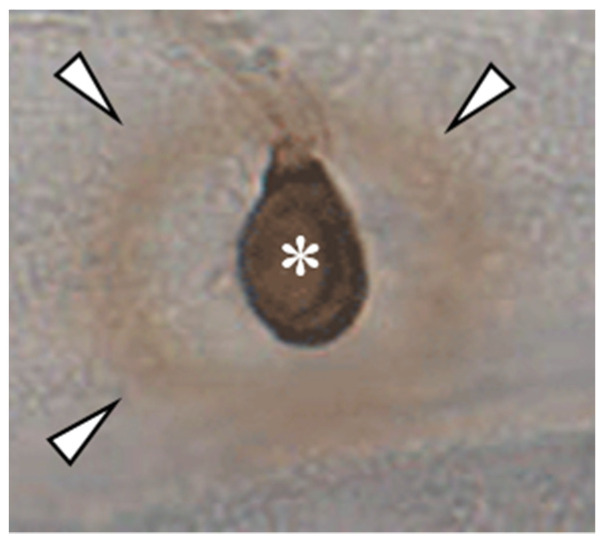
DAB-staining of an appressorium of *C. graminicola* on the epidermis of *Allium cepa*. Note the non-stained area between DAB-positive ring (arrowheads) and the appressorium (asterisk), representing an area devoid of H_2_O_2_.

Interestingly, melanin biosynthesis is rigorously stopped at appressorial maturation [43], raising questions regarding the task of highly accumulated appressorial osmolytes during host invasion and subsequent biotrophic development. Highly concentrated osmolytes such as mannitol and glycerol [38,48], as occurring in appressoria, are able to scavenge hydroxyl radicals [57,58], the formation of which belongs to the fastest plant defense responses [59]. Indeed, infection pegs that have breached the cell wall, are able to scavenge H_2_O_2_, as indicated by an unstained halo surrounding the invasion site after 3,3′-diaminobenzidine (DAB) staining. Outside of this halo, DAB caused brown staining of H_2_O_2_ (Figure 2). Interestingly, RNA-Seq studies have shown that genes encoding aquaglyceroporins are expressed in mature appressoria [3], and the corresponding proteins may serve to secrete ROS scavengers as soon as the infection peg extends from the appressorial base (Figure 2).

It would be interesting to generate mutants defective in those sugar alcohol transporters or aqua-glyceroporins that are expressed in appressoria and/or at the time of plant cell wall breaching. Additionally, it would be interesting to investigate whether mutants that constitutively form melanin would still be able to secrete sugar alcohols or glycerol from penetration pegs and to scavenge hydroxyl radicals.

### 3.2. Chitin and β-1,3-glucan Are Indispensable for Vegetative and Pathogenic Development

**Figure 3:**
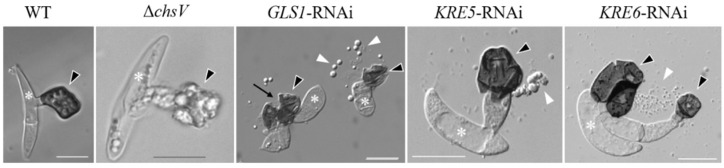
Appressoria formed by the *C. graminicola* WT strain, the *CHSV*-deficient mutant Δ*chsV*, and by *GLS1*-, *KRE5*-, and *KRE6*-RNAi-strains. White asterisks indicate conidia, black arrowheads appressoria, white arrowheads cell components released by ruptured appressoria, and the black arrow indicates an appressorial disruption site. Size bars are 10 µm. WT and *GLS1*-RNAi are from [63], *KRE5*- and *KRE6*-RNAi are from [64], and Δ*chsV* is from [61].

The presence of an average of eight chitin synthase (*CHS*) genes possibly executing overlapping functions, may be taken as an indirect indication of the importance of chitin in fungal cell walls. Indeed, the genome of *C. graminicola* harbors nine putative chitin synthase genes (Figure 1), [3]. In this fungus, a class VI *CHS* gene called *chsA* is essential for conidial cell wall strength in media with high water potential, as demonstrated in a disruption mutant [60]. RNA-Seq studies showed that three additional *CHS* genes of this fungus, i.e., *CHSI*, *CHSIII*, and *CHSV*, encoding class I, III, and V CHSs, respectively, are expressed during vegetative and pathogenic development (Figure 1) [3]. Deletion mutants lacking *CHSI* or *CHSIII* were comparable to the wild type with respect to vegetative growth and virulence. In contrast, mutants devoid of *CHSV*, encoding a class V chitin synthase protein (Chs) with an *N*-terminal myosin-like motor domain (MMD), are strongly impaired in both vegetative and pathogenic development [61]. Class V *CHS* genes exist in filamentous fungi but not in yeasts. In the maize smut fungus *Ustilago maydis*, Steinberg and co-workers fused a triple green fluorescent protein (GFP) tag to the class V chitin synthase *mcs1* and generated mutants lacking the MMD as well as mutants defective in actin binding or ATP hydrolysis. Collectively, infection assays, live cell imaging in combination with photobleaching, and Benomyl- and Latrunculin A-inhibitor studies suggested that the MMD of Mcs1 is required for short-range motility in the hyphal apex along F-actin, exocytosis, and virulence. Δ*mcs1* deletion mutants and mutants carrying amino acid exchanges interfering with chitin synthase activity of *U. maydis* Mcs1 exhibited severe hyphal swellings, indicating that this class V chitin synthase is crucial for hyphal morphology and virulence [62].

*C. graminicola* Δ*chsV* mutants exhibit large balloon-like swellings even in osmotically supported media, and vegetative hyphae contain intrahyphal hyphae [61]. While the WT strain differentiates melanized appressoria with rigid cell walls (Figure 3, WT, arrowhead), as discussed above, Δ*chsV* mutants formed appressorial initials with apparently normal cell walls, but, intriguingly, the cell walls of these infection cells lyse when appressoria mature, leaving sac-shaped cells on the plant surface (Figure 3, Δ*chsV*, arrowhead). Conceivably, the wall-deficient infection cells of Δ*chsV* mutants were unable to build up turgor pressure and plant infection failed [61]. These experiments clearly showed that, in spite of the fact that several *CHS* genes exist and are expressed simultaneously at distinct stages of the *C. graminicola* infection process of maize leaves, *CHSV*-defects are not complemented by one or several other *CHS* genes and the *CHSV* gene of this fungus, thus represents a pathogenicity factor.

However, as indicated above, branched β-glucan, like chitin, represents a structural determinant of the fungal cell wall. As β-1,3-glucan synthase genes (*GLS1*/*FKS1*) are single-copy genes and as deletion mutagenesis was not successful, this gene appeared to be indispensable. In spite of its essentiality, RNA interference (RNAi) allowed investigating the role of *GLS1* of *C. graminicola* [63]. *GLS1*-RNAi strains exhibited massive cell wall swellings in and incorporation of a dark pigment into cell walls of vegetative hyphae. Unlike appressoria of Δ*chsV* mutants, those of *GLS1*-RNAi strains formed a cell wall, but appressoria spontaneously ruptured (Figure 3, *GLS1*-RNAi, arrows) and released the appressorial cell contents onto the plant surface [63] (Figure 3, *GLS1*-RNAi, arrowheads). The fact that functional β-glucan occurs as a branched polysaccharide suggests that genes and enzymes required for branch formation would also be important for appressorial plant invasion. Indeed, two key genes in β-1,6-glucan formation, *KRE5* and *KRE6* (Figure 1), are single-copy genes in *C. graminicola* and cannot be deleted. *KRE5*- and *KRE6*-RNAi strains, however, formed distorted vegetative hyphae [64] and appressoria with defective cell walls (Figure 3; *KRE5*- and *KRE6*-RNAi). The release of cell contents from defective appressoria was clearly visible (Figure 3; *KRE5*- and *KRE6*-RNAi, white arrowheads). It is intelligible that all strains with cell wall-defects were unable to form functional appressoria and were non-pathogenic [61,63,64].

**Figure 4:**
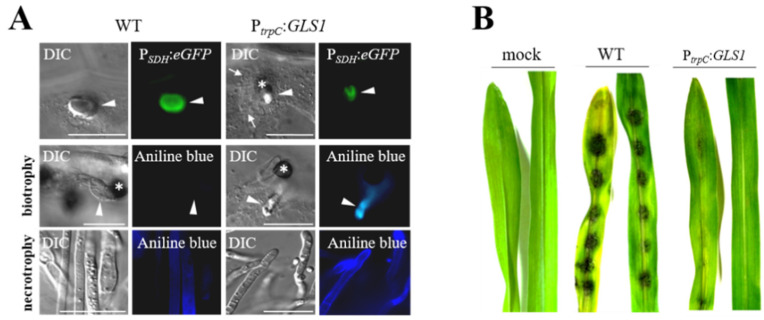
Forced expression of *GLS1* in biotrophic hyphae of *C. graminicola* affects hyphal morphology. (**A**) Comparison of biotrophic infection vesicles (top panel, arrowhead) formed by the WT strain and strains harboring an ectopically integrated *GLS1* copy controlled by the *trpC* promoter of *A. nidulans* shows that forced expression of *GLS1* caused reduction of the diameter of biotrophic hyphae. WT and P*_trpC_*:*GLS1* strains expressed *eGFP* under control of the biotrophy-specific saccharopine dehydrogenase promoter (P*_SDH_*:*eGFP*), confirming the biotrophic lifestyle of fluorescing hyphae. Aniline blue staining indicated the presence of β-1,3-glucan in cell walls of biotrophic hyphae of the P*_trpC_*:*GLS1* strain (biotrophy; aniline blue; P*_trpC_*:*GLS1*; arrowhead). No fluorescence was visible in biotrophic hyphae of the WT strain (biotrophy; aniline blue; WT; arrowhead). Necrotrophic hyphae of WT and P*_trpC_*:*GLS1* strain showed intense aniline blue staining (necrotrophy; aniline blue; WT and P*_trpC_*:*GLS1*; arrowhead). Asterisks indicate appressoria. DIC, differential interference contrast micrographs. Bars are 20 µm. (**B**) Disease symptoms on non-wounded maize leaves after inoculation with the WT strain or independent strains harboring a single ectopically integrated copy of *GLS1* controlled by the *trpC* promoter (P*_trpC_*:*GLS*). Mock-inoculated leaves were treated with 0.01% (*v/v*) Tween 20. From [63], modified.

RNA-Seq studies suggested that neither of the genes directly involved in branched β-glucan synthesis, i.e., *GLS1*, *KRE5*, and *KRE6*, nor any of the genes encoding β-glucan synthase-regulatory Rho proteins are differentially expressed (Figure 1). However, as indicated above, cell type-specific alterations in gene expression may remain undetected, due to poorly synchronized infection structure differentiation. Indeed, translational eGFP and mCherry fusions of *GLS1* and *KRE5* and *KRE6* revealed that these genes are not expressed or expressed below the detection limit of fluorescence microscopy in biotrophic infection vesicles and primary hyphae [63,64]. This observation is fully consistent with the idea that a PAMP release from these biotrophic hyphae, which are in close physical contact with PAMP receptor-loaded plasma membranes of maize, must be avoided. Thus, avoiding synthesis and surface exposure of branched β-glucan could be a strict requirement for circumventing PAMP-triggered immune responses (Figure 4A). However, we reasoned that strongly reduced formation of an important structural cell wall polymer such as branched β-1,3-glucan may be causal to the enormously increased volume of biotrophic hyphae, as observed not only in *C. graminicola*, but also in most other *Colletotrichum* species, in *M. oryzae*, and other hemibiotrophs [65,66]. Experimental evidence that reduced expression of β-1,3-glucan synthase and formation of β-glucan is causal for the increased volume of biotrophic hyphae came from constitutive *GLS1* expression experiments. Strains expressing *GLS1* under the strong constitutive *trpC* promoter of *Aspergillus nidulans* generated thin rather than voluminous biotrophic hyphae, and fluorescent Aniline Blue staining indicated that these strains produced and surface-exposed β-1,3-glucan (Figure 4) [63].

Disease resistance occurring against strains constitutively expressing *GLS1* was due to initiation of broad PTI responses, as indicated by RNA sequencing of inoculated maize leaves. In total, approx. 2180 genes showed more than 2.5-fold increased transcript abundances in leaves inoculated with P*_trpC_*:*GLS1* strains, several of which are known as PAMP-responsive genes, including genes encoding PR proteins, such as β-1,3-glucanases, chitinase, thaumatin- and germin-like proteins, cell wall reinforcing enzymes, and terpene synthases, possibly required for phytoalexin synthesis. In addition, transcripts of genes encoding serine-threonine receptor-like kinases, as well as zinc-finger and WRKY transcription factors were found increased [63]. Comparable broad induction of defense responses were also found in plants inoculated with strains constitutively expressing *KRE5* and *KRE6* genes [64].

The above studies [61,63,64] have demonstrated the structural role chitin and branched β-glucan in vegetative hyphae, appressoria, and infection hyphae of the hemibiotroph *C. graminicola*. However, with respect to severely reduced β-glucan contents in cell walls of biotrophic hyphae, the questions regarding the role of additional structural polymer(s) remain unanswered. Candidate polymers that might partially compensate the reduction of cell wall rigidity might be fungal-type galactomannan (FTGM). The α-core-mannan chain of FTGM consists of nine to ten sets of α-1,2-mannotetraose units connected by α-1,6-linkages, to which galactofuran side chains with β-1,5- and β-1,6-linked galactofuranosyl residues are connected via β-1,2-, β-1,3-, and/or β-1,6-bonds [67]. As FTGM is covalently connected to the chitin-β-1,3-glucan network via bonds to β-1,3-glucan and, furthermore, associated with plasma membranes via glycosylphosphatidylinositol (GPI) anchors, this polymer might play an important role in cell wall cross-linking and stability [67,68]. Highlighting the importance of this polymer in cell wall rigidity, mutants of *A. fumigatus* lacking either of the two core mannosyl transferases, CmsA or CmsB, as well as double mutants grow slowly and exhibit severe hyphal swellings [69]. Based on the severe reduction of β-1,3-glucan synthesis in biotrophic infection structures of *C. graminicola*, one may speculate that this polymer may be particularly relevant in infection structures with reduced contents of putatively PAMP-active structural cell wall polysaccharides.

### 3.3. Glycosylphosphatidylinositol (GPI) Anchor Biosynthesis Is Required for Hyphal Integrity and Pathogenicity

Covalent attachment of structurally complex glycolipids called glycosylphosphatidylinositol (GPIs) to the C-terminus of proteins is among the most common post-translational protein modifications in fungi [70]. Employing in silico analyses, Klis and co-workers identified 58 GPI-anchored proteins in *S. cerevisiae*, 20 of which representing putative plasma membrane proteins, and 38 potential cell wall proteins have been estimated [71]. In yeast and in *A. fumigatus*, GPI anchored proteins have been studied in detail, revealing that their distinct functions are central to the integrity of the cell wall. For example, members of the Crhp (Congo Red hypersensitive) protein family, originally discovered as required for the establishment of a linkage between chitin and β-1,6-glucan, have been identified as transglycosilases [72,73]. Two Gel/Gas proteins from *S. cerevisiae* and *A. fumigatus*, i.e., *Sc*Gas1p and *Af*Gel4p, exhibit dual β-1,3-glucan elongating and branching activity [74]; and Dfg (defective in filamentous growth) proteins, also carrying GPI residues, are required for inserting galactomannan into the cell wall [75]. Nine *DFG* genes are expressed during the infection process of *C. graminicola*, with distinct levels and timing of expression (Figure 1). Several GPI-anchored proteins are required in fungal virulence not only in *A. fumigatus* [11,76], but also in plant pathogenic fungi [77,78].

In *C. graminicola*, three key genes of GPI anchor biosynthesis, i.e., *GPI12*, *GPI8,* and *GAA1* have been identified and characterized. *GPI12* encodes a putative cytoplasmic *N*-acetylglucosaminylphosphatidylinositol deacetylase. *GPI8* and *GAA1* are catalytic components of the GPI transamidase complex. *GPI8* codes for a cystein protease involved in the proteolytic processing of the GPI anchoring signal at the C-terminus of the protein. *GAA1* encodes a putative M28 family-type metallo-peptide-synthetase probably required for formation of the peptide bond between the ω-site of a protein and a phosphoethanolamine group of the GPI lipid anchor [79,80]. Due to the central importance of the GPI biosynthetic pathway, attempts to delete the above-mentioned key genes by homologous recombination were unsuccessful, highlighting the essential character of these genes [81]. However, as shown for indispensable β-glucan synthesis genes above, RNAi approaches have been successfully applied, demonstrating that disturbance of GPI anchor biosynthesis had adverse effects on both vegetative and pathogenic development of *C. graminicola* [81]. It would be interesting to generate mutants lacking individual members of the above-mentioned classes of GPI-anchored cell wall proteins and to compare the virulence phenotypes in plant and human pathogenic fungi.

## 4. Hiding of PAMPs Contributes to the Establishment of Compatibility

PAMPs include a growing list of microbial molecules such as fungal chitin and branched β-glucans, ergosterol, and xylanases, as well as glycoproteins of oomycetes, and lipo-oligosaccharides of Gram-negative bacteria, bacterial flagellin. PAMPs are recognized by membrane-spanning pattern recognition receptors (PRRs), with the PAMP recognition domain facing the interaction site with a putative pathogen [82,83,84,85,86]. Using live-cell imaging, Zipfel and co-workers showed that the FLAGELLIN SENSING 2 receptor FLS2 formed 356 and 387 nm plasma membrane nanoclusters in epidermal cells of *Arabidopsis thaliana* and *Nicotiana benthamiana*, respectively, with high densities of approx. 2.2 FLS2 clusters per µm^2^ in either species [86]. Moreover, in ten plant species studied, a total of 201 genes encoding PRR proteins with chitin-binding LysM domains have been identified, suggesting that, as LysM genes are ubiquitous in the plant kingdom, uncountable LysM receptors with a complex domain architecture catalogue exist [87]. However, as in addition to chitin oligomers, oligomers of other invariable structural cell wall carbohydrates with PAMP activity are recognized, one may assume that numerous other PAMP receptors exist. Therefore, successful pathogens must have developed highly effective means of circumventing PAMP perception and activation of PTI in order to establish compatible interactions with their hosts.

At critical phases of the *Colletotrichum*-plant interaction, i.e., when a biotrophic infection hypha invades the epidermal plant cell, it becomes surrounded by the PRR-loaded plant plasma membrane, and PAMPs exposed on the fungal cell wall would immediately be detected. Furthermore, PAMP perception is supported by low-level constitutive secretion of plant chitinases and β-1,3-glucanases into the apoplast, which release elicitor-active fungal cell wall fragments that, in turn, are recognized by their respective PRRs. Thus, establishment of effective mechanisms hiding PAMP-active cell wall fragments is indispensable. This, as described below, can be accomplished in different ways, such as avoidance of PAMP formation, enzymatic conversion of elicitor-active cell wall polymers into molecules with reduced or no elicitor activity, masking by small secreted proteins with carbohydrate-binding activities (apoplastic effectors), and/or apposition of carbohydrate layers lacking elicitor activity onto surfaces of infection structures that contact the plant plasma membrane.

### 4.1. Obligatory Reduction of PAMP Synthesis during Biotrophy: Cell Type-Specific Transcriptional Regulation of Branched β-glucan Formation in C. graminicola

**Figure 5:**
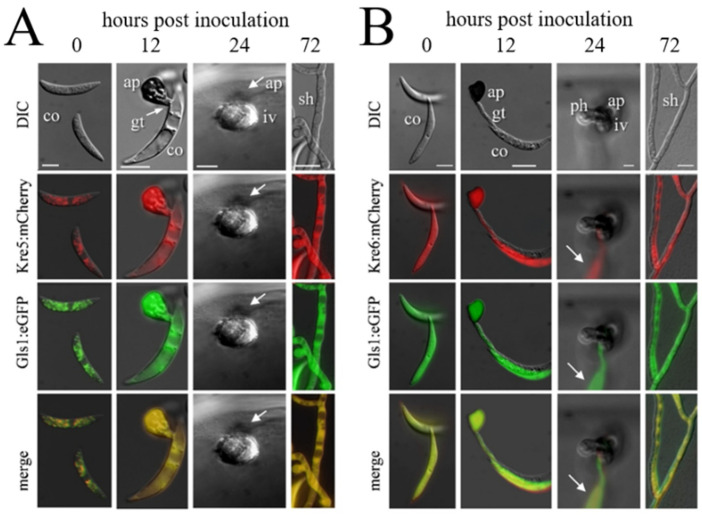
Infection structure-specific formation of Kre5:mCherry and Kre6:mCherry, and of the Gls1:eGFP fusion proteins. DIC, differential interference microscopy; Kre5:mCherry, Kre6:mCherry, and Gls1:eGFP show fluorescing protein in different infection structures. ap, appressorium; co, conidium; gt, germ tube; iv, infection vesicle; ph, primary hypha; sh, secondary hypha. Bars in A, B, and E are 10 µm. (**A**) Co-expression of *KRE5* and *GLS1*. (**B**) Co-expression of *KRE6* and *GLS1*. Note that no eGFP or mCherrc fluorescence is visible in biotrophic infection vesicles and primary hyphae (24 hpi). Arrows indicate a fluorescing conidium on the cuticle. From [64].

In fungal pathogens exhibiting a hemibiotrophic lifestyle, rigorous cell type-specific transcriptional down-regulation of PAMP formation may occur in biotrophic hyphae. As infection structure differentiation does not occur in a highly synchronized fashion, RNA sequencing data may not depict the lack of transcripts in individual cells (compare Figure 1 and Figure 5). Therefore Oliveira-Garcia and Deising generated translational Gls1:eGFP, Kre5:mCherry, and Kre6:mCherry strains and employed these in maize infection assays [63,64]. While both green eGFP fluorescence indicative of *GLS1* expression and red mCherry fluorescence showing *KRE5* and *KRE6* expression were visible in non-germinated conidia, germ tubes, and appressoria, fluorescence disappeared in biotrophic infection vesicles and primary hyphae (Figure 5). At later stages, i.e., at 72 hpi, when a lifestyle switch to necrotrophy had occurred, eGFP and mCherry fluorescence were clearly restored, indicating *GLS1*, *KRE5*, and *KRE6* expression at that stage of the interaction. These data suggest that, with the exception of biotrophic hyphae, β-1,3- and β-1,6-glucan biosynthesis occurs and that branched β-glucan is formed and surface-exposed in all cell types and lifestyles analyzed (Figure 5). Indeed, the cell type-specific expression profiles of *GLS1*, *KRE5*, and *KRE6* strongly suggest that reduction of gene expression, synthesis of enzymes, and PAMP-active branched β-glucans were reduced in order to avoid activation of PTI [63,64].

The requirement for biotrophy-specific down-regulation of *GLS1*, *KRE5*, and *KRE6* expression for successful colonization of maize epidermal cells was tested by constructing strains expressing these genes under the control of the strong constitutive *TrpC* promoter of *A. nidulans* or the *ToxB* promoter of the wheat pathogen *Pyrenophora tritici-repentis* [63,64]. Intriguingly, both promoters mediated formation of β-1,3- and β-1,6-glucan in biotrophic hyphae and, as a result, massive activation of PAMP responsive defense genes, e.g., genes encoding PR-proteins, plant cell wall synthesis enzymes, as well as terpene synthases likely required for phytoalexin biosynthesis [63,64,88].

Collectively, live cell imaging together with forced constitutive expression of *GLS1*, *KRE5*, and *KRE6* showed that transcriptional down-regulation of PAMP synthesis is required for the establishment of compatibility in *C. graminicola*-infected maize leaves.

### 4.2. Enzymatic Deacetylation of Chitin

Like branched β-glucan, chitin represents a cell wall scaffold and, at the same time, PAMP-active chitin fragments may be liberated from hyphal surfaces by plant chitinases and trigger defense responses at nanomolar concentrations [89]. Labelling of infection structures of the wheat stem rust and broad bean rust fungi *P. graminis* f. sp. *tritici* and *Uromyces viciae-fabae*, and of the maize anthracnose fungus *C. graminicola* with fluorescent Wheat Germ Agglutinin and antibodies to chitosan, i.e., *N*-deacetylated chitin, suggested that chitin was enzymatically converted to chitosan (poly-glucosamine) by secreted chitin deacetylases (Cdas) (Figure 1) [90]. Biotrophic hyphae showed chitin only at the very tip of the hypha, in agreement with the assumption that some time is required for this enzymatic conversion. Surface modification by chitin de-*N*-acetylation is thought to protect fungal cell walls from host chitinases and thus from generation of PAMP-actice chitin fragments initiating PTI in the invaded host tissue [90]. However, in the hemibiotrophic rice blast fungus *M. oryzae,* chitin deacetylases-defective mutants were not affected in pathogenic development [91,92]. Moreover, the biotrophic maize smut fungus *U. maydis* harbors a family of six active chitin deacetylase genes and one pseudogene. Of the six active genes, five were GPI-anchored, and one was a secreted soluble enzyme. One of the GPI-anchored Cdas, Cda7, belongs to a novel class of fungal Cdas [93]. Importantly, single as well as all higher order mutants lacking Cda7 had virulence defects. The secreted soluble Cda4 was implicated in deacetylation of the chitin surface layer and production of the chitosan layer surrounding biotrophic hyphae. However, in contrast to the hypothesis that surface-localized chitosan would protect hyphae from chitinase attack and suppress defense responses, the loss of this layer did not reduce virulence of *U. maydis* Δ*cda4* mutants. Attempts to inactivate all *CDA* genes simultaneously were unsuccessful, suggesting an essential role of chitosan for cell wall integrity [93]. Interestingly, strengthening the role of fungal Cdas in plant infection, the maize mannose-binding cysteine-rich receptor-like secreted protein (CRRSP)-AFP1 binds mannosylated Cdas, inhibits Cda activity, and exhibits antifungal activity [94].

### 4.3. Secretion of Cell Wall Polymer-Binding Effectors

The genomes of *Colletotrichum* species harbor large numbers of genes coding for putative secreted effectors, which, in most cases, are small, cysteine-rich proteins lacking homology to proteins outside the genus *Colletotrichum* [3]. Comprehensive genome and transcriptome analyses in *C. graminicola* and *C. higginsianum* suggested the presence of 177 putative effector genes in *C. graminicola*, and twice as many, namely 365, in *C. higginsianum* [3]; see also [95]. Employing a method called the yeast signal sequence trap (YSST), 103 unigenes encoding putative effectors were identified in *C. graminicola*. Fifty of these showed similarities to genes with a known function, 25 showed similarity to genes without a known function, and 28 showed no similarity to entries in any database [96]. Macro array and quantitative reverse-transcriptase polymerase chain reaction analyses confirmed that most of the genes identified by the YSST screen are expressed during maize infection, and a large set showed peaks of transcript abundances at specific phases of pathogenesis [96]. Likewise, in *C. higginsianum*, deep sequencing of the transcriptome at infection stages representing distinct lifestyles identified sequential delivery of effectors, with different antagonistic effectors inducing or suppressing plant cell death [97]. These authors concluded that hemibiotrophy in *C. higginsianum* is orchestrated through the coordinated expression and secretion of effector waves. It should be emphasized that expression and function of effectors genes has not only been analyzed in *Colletotrichum* species [65,98,99,100,101,102], but also in several other fungal pathogens [103,104,105,106].

Intriguingly, some of the apoplastic effectors exhibit cell wall polymer-binding activities and have proven effects on fungus–host interactions. One of the first effector genes of the biotrophic tomato pathogen *Cladosporium fulvum*, *Avr4*, encodes the chitin-binding effector protein Avr4 [107,108]. The fact that pathogenic fungi employ secreted effectors to bind to invariable cell wall polymers to inhibit liberation and recognition of PAMPs by their respective PRRs is plausible. Indeed, the chitin-binding LysM effectors Avr4 and Ecp6 of *C. fulvum* both interfere with PTI. Whereas Avr4 binds to fungal hyphae and protects them from chitinase attack, Ecp6 sequesters tri-, penta-, and hexameric chitin fragments and turns them undetectable for plant LysM receptors, making the Avr4-Ecp6 effector interplay ideally suited to compromise defense responses [109].

**Figure 6:**
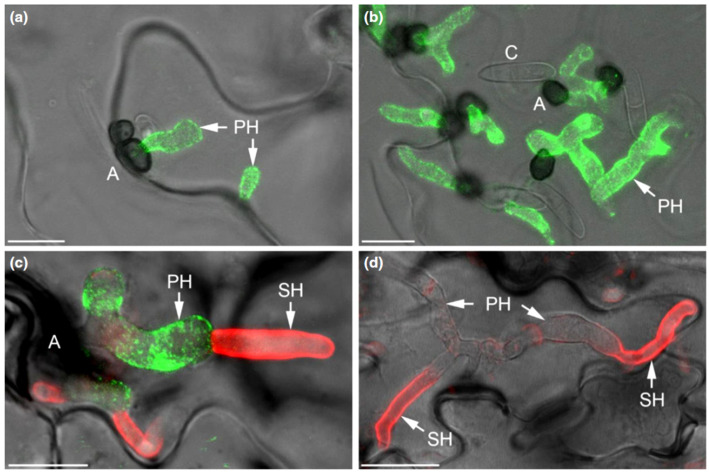
The *C. higginsianum* extracellular LysM protein ChELP2 is localized at the biotrophic interface. (**a**–**d**) Confocal micrographs showing the localization of ChELP2 (green) and chitin (red) in *C. higginsianum*-infected Arabidopsis leaf tissue. ChELP2 was detected by the monoclonal antibody UB25 exclusively on biotrophic primary hyphae (**a**–**c**), and chitin, as labeled with wheat germ agglutinin (WGA), was recognized only on the surface of necrotrophic secondary hyphae (**d**), but not primary hyphae. Appressoria, A; conidia, C; primary hyphae, PH; secondary hyphae, SH. Bars are 10 µm. From: [110]. Reprinted with permission from Richard O’Connell and with permission of John Wiley and Sons, license number 5265240188317.

The genomes of the hemibiotrophic Arabidopsis- and maize-infecting pathogens *C. higginsianum* and *C. graminicola* harbor a large repertoire of candidate-secreted effectors containing LysM domains, i.e., 18 and 14 genes, respectively [110] (Figure 1). Two of the LysM effectors of *C. higginsianum*, ChELP1 and ChELP2, represent homologs of CIH1 (for *Colletotrichum* intracellular hypha 1) of the bean anthracnose fungus *C. lindemuthianum* [111]. CIH1 was initially identified as a proline-rich glycoprotein of the cell wall and the interfacial matrix, separating the fungal cell wall from the plasma membrane of the invaded host [111], and later recognized as a LysM domain protein [110]. Immuno-cytochemistry showed that ChELP2 localizes to the surface of biotrophic hyphae, i.e., at the interface with living host cells (Figure 6). Both, ChELP1 and ChELP2, bind to chitin and chitin oligomers with high affinity, and both proteins suppress the chitin-triggered activation of two defense-related plant mitogen-activated protein kinases in *A. thaliana*. Importantly, ChELP1 and ChELP2 are both required for appressorium-mediated host invasion and full virulence [110].

Indeed, Ecp6 orthologs are present in many fungi and often occur as multigene families [112]. The secreted LysM effector Mg1LcsM of the wheat pathogen *Zymoseptoria tritici* protects fungal hyphae through chitin-dependent homodimer polymerization [113], and homodimer polymerization may mechanistically be required for protection of hyphae of other fungi as well. Binding of the *C. fulvum* LysM effectors to fungal cell walls has been visualized, employing fluorescence conjugates of Ecp6 or Avr4 [113].

Taken together, LysM motifs are present in plant PRRs recognizing chitin (see above), as well as in fungal apoplast-localized chitin-binding effectors, with distinct functions of plant and fungal LysM proteins in detecting of pathogen attack and masking the hyphal surface [114].

Not only chitin-binding LysM effectors, but also other proteins with carbohydrate polymer-binding specificities have been identified. The basidiomycete and root endophyte, *Piriformospora indica*, colonizing different host plants, including barley and *A. thaliana*, secretes a fungal β-glucan-binding lectin (FGB1), which efficiently suppresses β-glucan-triggered defense responses in different hosts [115,116]. Genes encoding β-glucan-binding proteins with similarities to FGB1 have not been found in the genome of *C. graminicola* (Alga Zuccaro, personal communication), suggesting that reduction of β-glucan synthesis and formation β-glucan-binding proteins may represent alternative mechanisms to evade defense responses.

### 4.4. Cell Surface Appositions Putatively Function in Compatibility

As an alternative or in addition to hyphal masking by fungal carbohydrate-binding effector proteins, apposition of non-elicitor-active cell wall polymers has been proposed. Evidence for PAMP-masking by cell wall appositions was provided by alkali-treatment of infection structures of the broad bean rust fungus *U. viciae-fabae*. Removal of alkali-soluble polymers, primarily consisting of α-1,3-glucan, resulted in surface exposure of chitin, as indicated by strong wheat germ agglutinin (WGA) labelling [117]. Since α-1,3-glucan is not degradable in plants, one may argue that fungal plant pathogens use α-1,3-glucan as a mechanism to bypass PTI [26]. Accordingly, Fujikawa and co-workers reported that the rice blast fungus *M. oryzae*, like the rust fungus, masks its cell wall surfaces by α-1,3-glucan apposition in order to not expose PAMP-active cell wall polymers [26]. Indeed, rice leaves inoculated with Δ*ags1* mutants of *M. oryzae* defective in the single-copy α-1,3-glucan synthase gene did not develop any disease symptoms, and the authors interpreted this as a result of lacking PAMP-masking and induction of defense responses. Surprisingly, however, infection structures formed by this mutant lysed on the leaf surface already at 10 hpi, long before mature appressoria are formed and an invasion of the host takes place. These data are inconsistent with the idea that plant defense responses would be responsible for hyphal lysis. Moreover, RT-qPCR analyses only revealed a moderate transcriptional defense response of plants to Δ*ags1* mutants. As compared with the WT strain, the transcript abundances of three rice defense genes analyzed, i.e., *OsPR1a*, *OsPR3*, and *OsPBZ1* were reported as 2-, 2.4-, and 1.7-fold increased at 10 hpi [26]. To illustrate how strong PAMP-dependent transcriptional responses can be, *GLS1*-overexpression strains of *C. graminicola* may serve as a reference [63]. In maize plants inoculated with those strains, more than 2000 genes showed a 2.5-fold or higher increase in transcript abundances, with genes encoding classical PAMP-response proteins such as β-1,3-glucanases, a germin-like protein, plant cell wall-modifying enzymes, terpene synthases, receptor-like serine-treonine kinases, or WRKY transcription factors, showing up to 1000-fold increased transcript abundances [63]. Comparably dramatic responses were observed in maize leaves inoculated with *C. graminicola* strains overexpressing the key β-1,6-glucan synthesis genes *KRE5* and *KRE6* [64].

*A. fumigatus* mutants lacking all three *AGS* genes exhibited a layer of ConA-positive glycoproteins on the surface rodlet layer, most of which were identified as hydrolases. These data strongly suggest that the lack of α-1,3-glucan had led to reduced cell wall retainment of these proteins [118]. An increase in the motility of cell wall-modifying enzymes, due to lack α-1,3-glucan and, therefore, to increased cell wall pore diameters may be fully sufficient to explain lysis of infection structures of *M. oryzae* Δ*ags1* mutants on the plant surface, as observed by Fujikawa et al. [26]. These considerations raise doubts regarding the function of α-1,3-glucan as a PAMP concealing cell wall apposition.

## 5. Conclusions

**Figure 7:**
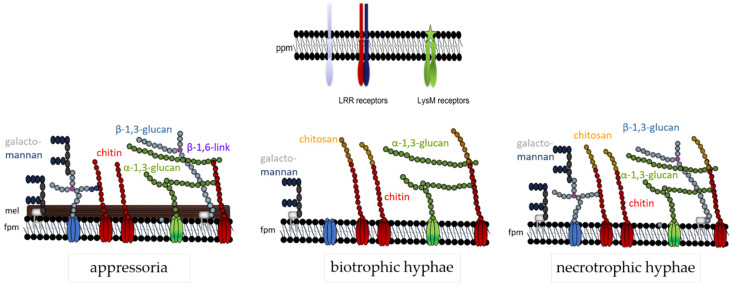
Cell wall model of *C. graminicola* at three different developmental stages. The fungal plasma membrane (fpm) harbors cell wall polymer-synthesizing enzymes, e.g., chitin synthases (red), α-1,3- (green) or β-1,3-glucan synthases (light blue). Chitin (red spheres), β-(1,3)-glucan (light blue spheres) with β-(1,6)-branching sites (purple spheres), and galactomannans (blue spheres), represent structural polymers, whereas and α-(1,3)-glucan (green spheres) is a mucoid polym. Proteins and galactomannans may be GPI-anchored. Note that the cell wall structure is lifestyle-dependent: appressoria possess a melanin layer (mel) and strong layers of structural carbohydrate polymers. Biotrophic hyphae are surrounded by the plant plasma membrane (ppm) containing PAMP receptors, and thus lack β-glucan. In biotrophic hyphae, surface-localized chitin is enzymatically converted to chitosan (yellow spheres). Necrotrophic hyphae exhibit all the polymers described. After [63], modified.

The data discussed here indicate that the fungal cell wall is not a static, but rather a highly dynamic structure, and this may be particularly true of plant pathogenic fungi exhibiting a biotrophic or hemibiotrophic lifestyle. In these pathogens, cell wall biogenesis must respond to the host environment, and the presence of plant membrane-localized PRRs requires masking of those cell wall polymers that exhibit PAMP activity. Thus, in *C. graminicola* appressorial cell walls, all polymers providing cell wall rigidity are formed. At that developmental stage, PAMP exposure is probably less critical. In addition, as visualized by dark pigmentation, melanin is incorporated in direct vicinity to the fungal plasma membrane. At critical phases of the interaction, however, i.e., when biotrophic infection structures face plant PRRs, formation of β-glucan is strongly and cell-specifically reduced, and chitin is deacetylated, possibly to avoid cleavage by chitinases and liberation of oligomeric PAMPs. In addition, indicative of the importance of avoidance of chitin-based PAMP detection, LysM effectors are present and likely secreted at that stage of development (Figure 1). After the switch to necrotrophy, however, synthesis and surface-exposure of PAMP-active cell wall polymers is re-established. At that stage of infection, plant cells are killed and defense responses will likely vanish. A summary of the cell wall dynamics in infection cells of *C. graminicola* is given in Figure 7.
